# Effects of doxorubicin associated with amniotic membrane stem cells in the treatment of canine inflammatory breast carcinoma (IPC-366) cells

**DOI:** 10.1186/s12917-020-02576-0

**Published:** 2020-09-24

**Authors:** Jéssica Borghesi, Sara Caceres, Lara Carolina Mario, Angela Alonso-Diez, Ana Carolina Silveira Rabelo, Maria J. Illera, Gema Silvan, Maria Angélica Miglino, Phelipe O. Favaron, Ana Claudia O. Carreira, Juan Carlos Illera

**Affiliations:** 1 Department of Surgery, School of Veterinary Medicine and Animal Science, University of Sao Paulo, Sao Paulo, Brazil; 2 Department of Animal Physiology, School of Veterinary Medicine, Complutense University of Madrid (UCM), Madrid, Spain; 3 Department of Animal Medicine, Surgery and Pathology, School of Veterinary Medicine, Complutense University of Madrid (UCM), Madrid, Spain; 4 NUCEL (Cell and Molecular Therapy Center), School of Medicine, University of Sao Paulo, Sao Paulo, Brazil

**Keywords:** Amniotic membrane, Breast cancer, Co-culture, Dog, Therapy

## Abstract

**Background:**

Tumours in mammary glands represent the most common neoplasia in bitches, as in humans. This high incidence results in part from the stimulation of sex hormones on these glands. Among mammary tumours, inflammatory carcinoma is the most aggressive, presenting a poor prognosis to surgical treatment and chemotherapy. One of the most widely used chemotherapy drugs for breast cancer treatment is doxorubicin (DOXO). Alternative therapies have been introduced in order to assist in these treatments; studies on treatments using stem cells have emerged, since they have anti-inflammatory and immunomodulatory properties. The aim of this study was to evaluate the effects of DOXO and canine amniotic membrane stem cells (AMCs) on the triple-negative canine inflammatory mammary carcinoma cell line IPC-366.

**Methods:**

Four experimental groups were analysed: a control group without treatment; Group I with DOXO, Group II with AMC and Group III with an association of DOXO and AMCs. We performed the MTT assay with DOXO in order to select the best concentration for the experiments. The growth curve was performed with all groups (I-III) in order to verify the potential of treatments to reduce the growth of IPC-366. For the cell cycle, all groups (I-III) were tested using propidium iodide. While in the flow cytometry, antibodies to progesterone receptor (PR), estrogen receptor (ER), PCNA, VEGF, IL-10 and TGF-β1 were used. For steroidogenic pathway hormones, an ELISA assay was performed.

**Results:**

The results showed that cells treated with 10 µg/mL DOXO showed a 71.64% reduction in cellular growth after 72 h of treatment. Reductions in the expression of VEGF and PCNA-3 were observed by flow cytometry in all treatments when compared to the control. The intracellular levels of ERs were also significantly increased in Group III (4.67% vs. 27.1%). Regarding to the levels of steroid hormones, significant increases in the levels of estradiol (E2) and estrone sulphate (S04E1) were observed in Groups I and III. On the other hand, Group II did not show differences in steroid hormone levels in relation to the control. We conclude that the association of DOXO with AMCs (Group III) promoted a reduction in cell growth and in the expression of proteins related to proliferation and angiogenesis in IPC-366 triple-negative cells.

**Conclusions:**

This treatment promoted ER positive expression, suggesting that the accumulated oestrogen conducted these cells to a synergistic state, rendering these tumour cells responsive to ERs and susceptible to new hormonal cancer therapies.

## Background

The development of tumour cells arises from a series of sequential mutations resulting from genetic instability. A more recent study has shown that the influence of environmental factors on healthy cells can generate these mutations, resulting in the formation of tumours [1].

Tumours in canine mammary glands represent the most common neoplasm in female dogs [2]. These types of tumours in female dogs are very similar to tumours presented in humans regarding their biological behaviour, their response to cytotoxic agents and their histological characteristics, but their incidence rate is three times higher than in women [3]. This high incidence results in part from the stimulation by sex hormones of the mammary glands of female dogs and women exposed to them during their reproductive life [4]. This hypothesis is supported by the expression of estrogen and progesterone receptors; it is possible that they act as promoting factors, stimulating cell proliferation, but not acting as initiating factors. However, when acting together in combination with other factors, for example, environmental and epigenetic factors at different stages of tumour formation, it is possible that they play a determining role in tumour formation [5].

Among breast tumours, we can highlight inflammatory breast carcinoma (IBC) as the most aggressive neoplasm found, affecting both women and female dogs [6]. Inflammatory breast cancer is characterised pathologically by rapid progression. Although this cancer is the less frequent type of breast neoplasia, representing only 2–4% of total breast cancers in the US, it has the highest mortality rate [7]. Nowadays, triple-negative breast carcinoma remains a major challenge for both human and veterinary medicine in terms of diagnosis and treatment [8]. Caceres et al. 2015 [9] established the first canine inflammatory mammary carcinoma cell line, named IPC-366, with an epithelial phenotype and the triple-negative expressions of estrogen receptor (ER), progesterone receptor (PR) and human epidermal growth factor receptor 2 (HER-2). This newly established cell line maintains the same features *in vivo* and exhibits vasculogenic mimicry properties *in vitro* and *in vivo*. Usually, mammary cancer treatment in canine species is based on removal of the tumour and mammary chain, followed by adjuvant chemotherapy after surgical treatment in order to eradicate possible micrometastases and increase the survival of the animal [10]. However, this treatment is contraindicated in cases of inflammatory carcinoma due to the extensive cutaneous involvement and its association with coagulopathies [11]. Nevertheless, conventional chemotherapy alone is not effective. Authors have been studying alternative treatments of canine mammary cancer, such as the association of the drug doxorubicin (DOXO) with peroxidam [12]. Many other agents are used in other tumour types, such as the association of stem cells, which can reduce the size of the tumour or extend the survival of the organism [13].

Recently, there has been an increasing interest in stem cell studies, specifically of foetal stem cells. These cells have a great capacity for proliferation and expansion *in vitro* [14]. Furthermore, due the fact that they are found in the foetal–maternal interface, they are immunologically tolerated, making them a safe choice for use in transplants and cell therapy. Several transplant and graft studies have been performed with human amniotic membrane at term, and their results have demonstrated that these cells do not cause an immune response [15]. This lack of immunogenicity can be explained by the immunomodulatory properties possessed by foetal membranes, which are involved in maternal-foetal maintenance and tolerance [16]. Several mechanisms assist these characteristics, such as the function of the amniotic membrane to secrete anti-inflammatory proteins and its pro-apoptotic activity that promotes leukocyte apoptosis [17].

Consequently, the aim of this study was to evaluate the efficiency of amniotic membrane stem cells in association with drug treatments in canine mammary inflammatory carcinoma cell line.

## Methods

### Canine inflammatory mammary carcinoma cell line IPC-366

IPC-366 was obtained from the Department of Physiology of the Faculty of Veterinary Medicine of the Universidad Complutense de Madrid, which was previously characterised by Caceres et al. 2015 [9]. The cells were cultured in Dulbecco’s Modified Eagle Medium Nutrient Mixture F-12 Ham (DMEM/F12; Sigma-Aldrich, D6421) supplemented with 5% foetal bovine serum (FBS; Sigma-Aldrich, 12103C), 1% penicillin-streptomycin (Sigma-Aldrich, P0781) and 1% L-glutamine (Sigma-Aldrich, G7513), in 25 cm³ flasks and maintained at 37 ºC, with relative humidity close to 100% and a gas atmosphere of 5% CO_2_.

### Culture of the canine amniotic membrane stem cells

The canine amniotic membrane stem cells (AMCs) were obtained in a neutering campaign by the collection of pregnant uterus during hysterectomy, as approved by the Ethics Committee for Animal Use (CEUA) School of Veterinary Medicine and Animal Science, University of Sao Paulo, FMVZ-USP: PROEX329/15. Cell isolation was carried out according to Uranio et al. 2011 [18] and Park et al., 2012 [19]. These cells had previously been characterised by Borghesi et al. 2019 [20]. Cell culture was carried out at the FMVZ-USP. The AMCs were maintained in the same IPC-366 medium and culture conditions.

### Interaction assay through cell co-culture

For the interaction assay co-culture, the cells were seeded in 6-well transwell plates (Corning Inc, NY, USA). The AMCs (3 × 10^4^ cells) were seeded into the upper chamber of the transwells, while the IPC-366 cells (5 × 10^4^ cells) were incubated in the lower compartments. Cell treatments were carried out at 24, 48 and 72 h and divided into three experimental groups: Group I, IPC-366 treated with DOXO (10 µg/mL); Group II, IPC-366 treated with AMCs; and Group III, IPC-366 treated with an association of DOXO (10 µg/mL) and AMCs. A control group of IPC-366 cells without treatment was used in all experiments.

### Doxorubicin dosage determination

To determine sensitivity of IPC-366 cells to the cytotoxic effect of DOXO (Sigma-Aldrich, D1515), four different final concentrations, 1, 2, 5 and 10 µg/mL, were used to determine the critical concentration to be used in the MTT assays.

### Cell metabolism assay (MTT)

A total of 1 × 10³ IPC-366 cells per well were seeded in 96-well plates and, after their adhesion, different concentrations of DOXO were added. MTT analysis was performed after 24, 48 and 72 h. On each day of experiment, the culture medium was removed, then 10 µL of MTT solution (M5655, Sigma) was added and incubated for 2 h at 37 ºC in the dark. After this period, MTT solution was aspirated from the wells and the remaining formazan crystals dissolved in 100 µL of dimethyl sulfoxide (DMSO) (Sigma, M5655) and the absorbance measured at 490 nm in a spectrophotometer (MQuant– Bio Tek Instruments, VT, USA).

### Morphological analysis

For the morphological analysis cells were observed under an inverted microscope (NIKON ECLIPSE TS-100) every day of treatment, in order to verify the morphological changes.

### Growth curve

Approximately 2 × 10^4^ IPC-366 cells were cultured in 6-well plates (CLS3335, Sigma), and treated for 72 h. Every 24 h, three wells of each treatment and three control wells were counted with trypan blue to evaluate the cell growth.

### Cell cycle

For cell cycle analysis, cells were subcultured, washed with phosphate saline buffer (PBS) and fixed in 70% ethanol overnight at 4 °C. Cells were then treated with 40 mg/mL of propidium iodide (PI), 100 ug/mL (RNaseA, Sigma) and the samples analysed in a flow cytometer (FACSAriaII Cell Sorter, Becton Dickinson, San Jose, California, USA) and analysed by software Modfit 2.9.

### Flow cytometry

Immunophenotypic analysis was performed using flow cytometry. For this, 1 × 10^5^ cells/mL were trypsinised, centrifuged and fixed in 4% of paraformaldehyde. The cells were then incubated with primary antibodies (dilution 1:100) for 30 min at 4 °C. After this period, the cells were incubated with the secondary antibody (dilution 1:500) for 30 min at 4 °C. Finally, the cells were washed and analysed by a BD FACSAriaII Flow Cytometer (Becton Dickinson, San Jose, CA, USA). Controls were performed using unmarked cells exposed only to the non-specific secondary antibody. For each sample, 10,000 events were counted. The primary antibodies used were as follows: Anti-VEGF (ab1316, Abcam, Cambridge, UK), Anti-PCNA-3 (sc-46, Santa Cruz Biotechnology Inc, Europe), Anti-PR (ab-2764, Abcam, Cambridge, UK), anti-ER (ab2746, Abcam, Cambridge, UK), anti-TGF-β1 (sc-146, Santa Cruz Biotechnology Inc, Europe) and anti-IL-10 (MCA2111B, AbD Serotec, California, USA). The secondary antibodies used were anti-mouse (ab150115, Abcam, Cambridge, UK) and anti-rabbit (ab150079, Abcam, Cambridge, UK), and analysed by software FlowJo 10.

### Steroid hormone analysis

The culture medium of control and treated cells were collected and frozen at ‑20 °C until hormone analysis. The levels of hormones were measured by immunoenzymatic assay (ELISA), previously validated by Illera et al., 2016 [21]. Steroid hormones tested were estrone sulphate (SO4E1: ab R522-2), estradiol (E2: C6E91), androstenedione (A4: ab C9111), testosterone (T: R156), dihydrotestosterone (DHT: C1D4), dehydropeiandrosterone (DHEA: C9411), progesterone (P4: C914) and pregnenolone (P5: C1P53). All antibodies were developed in the Department of Animal Physiology (UCM, Spain). All hormone concentrations are expressed in ng/mL. Three independent experiments were performed.

### Statistical analysis

The results obtained from the hormonal analysis were submitted to the two-way ANOVA followed by the Bonferroni post-hoc test. For this analysis, the software used was Graph Pad Prism version 5.0. Statistically significant differences were considered when *p* < 0.05.

## Results

### Doxorubicin treatment on proliferation of IPC-366 cells

**Fig. 1:**
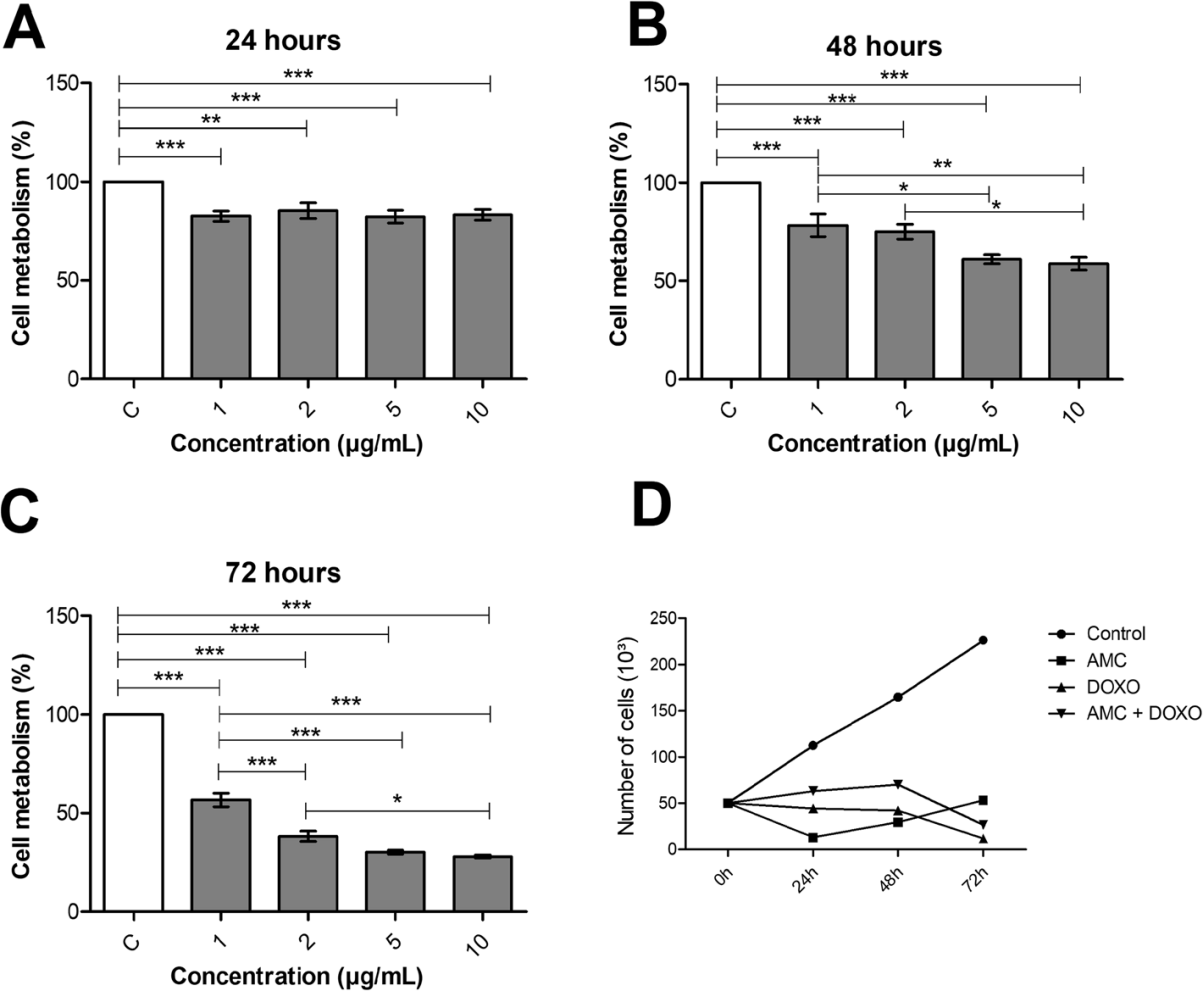
Cell metabolism assay by MTT analysis of IPC-366 control cells and those treated with 1–10 µg/mL of DOXO for 24 (**a**), 48 (**b**) and 72 h (**c**). Growth curve analysis was evaluated in 24 h, 48 h, and 72 h (**d**). DOXO: doxorubicin; AMCs: amniotic membrane cells. Data are averaged (*n* ≥ 04 ± SEM), performed with one-way analysis of variance (ANOVA) as a Bonferroni pos-hoct test, *p < 0.05, **p < 0.01, ***p < 0.001

To establish the optimal chemotherapeutic dosage of IPC-366 cells, distinct concentrations of DOXO (1, 2, 5 and 10 µg/mL) were tested by the MTT colorimetric method. The 10 µg/mL dosage showed the highest efficacy in the treatment of IPC-366, with a reduction of 22.76% after 24 h, 50.76% at 48 h and 71.64% at 72 h. (Fig. 1a-c). Therefore 10 µg/mL dosage was chosen to be used during treatment.

### Analysis of growth curve and morphology

First of all, cell growth was observed through the growth curve. In the Group I, the cells showed a continuous decrease in growth, differing from the control group. Although Group II showed cell growth over 72 h, there was still less growth than the control group. In Group III, the growth curve showed discrete growth until 48 h compared to control. After this period, there was a decline in growth (Fig. 1d).

**Fig. 2:**
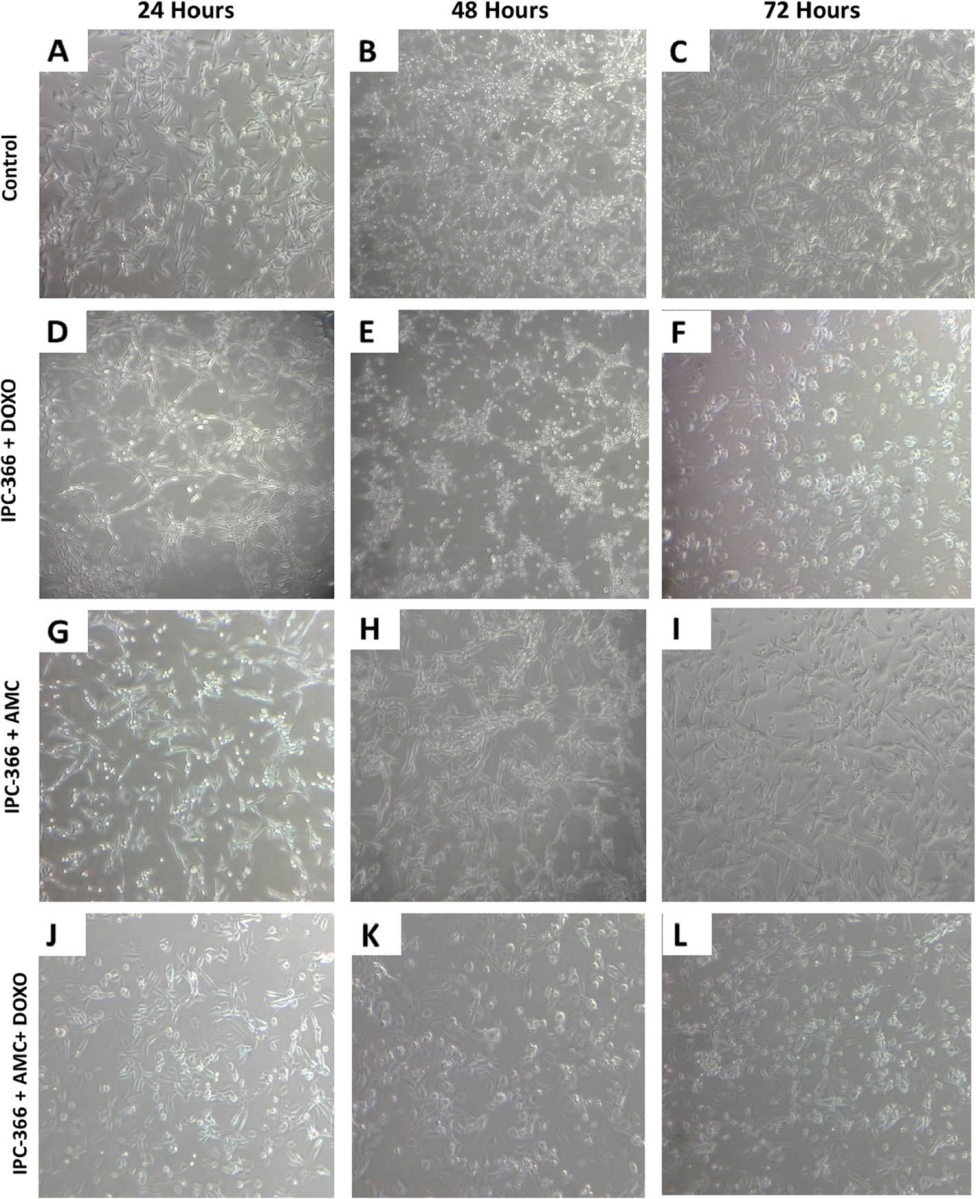
Morphological analysis of IPC-366 cells over 72 h of treatment. Images were taken at 5 × magnification. **a-c** IPC-366 control cells; **d-f** IPC-366 treated with DOXO (10 µg/mL), **g-i** IPC-366 treated with AMCs; **j-l** IPC-366 treated with AMCs and DOXO (10 µg/mL)

Morphological changes in the cells during the different treatments were also observed. Analysing the control cells during the 72-h period, the epithelial shape remained and a confluence, with the appearance of interlaced cells, formed a cellular cluster (Fig. 2a-c). Group I at 24 h maintained the same epithelial morphology as control; however, after 48 h the cells lost their original shape becoming rounded and irregular (Fig. 2d-f). In Group II, cells maintained their epithelial morphology with the same characteristics as the control during treatment (Fig. 2g-i). In Group III after 48 h only a few cells began to lose their original morphology, differing from Group I, where virtually all cells lost their original format, showing an irregular shape (Fig. 2j-l).

### Cell cycle analysis

**Fig. 3:**
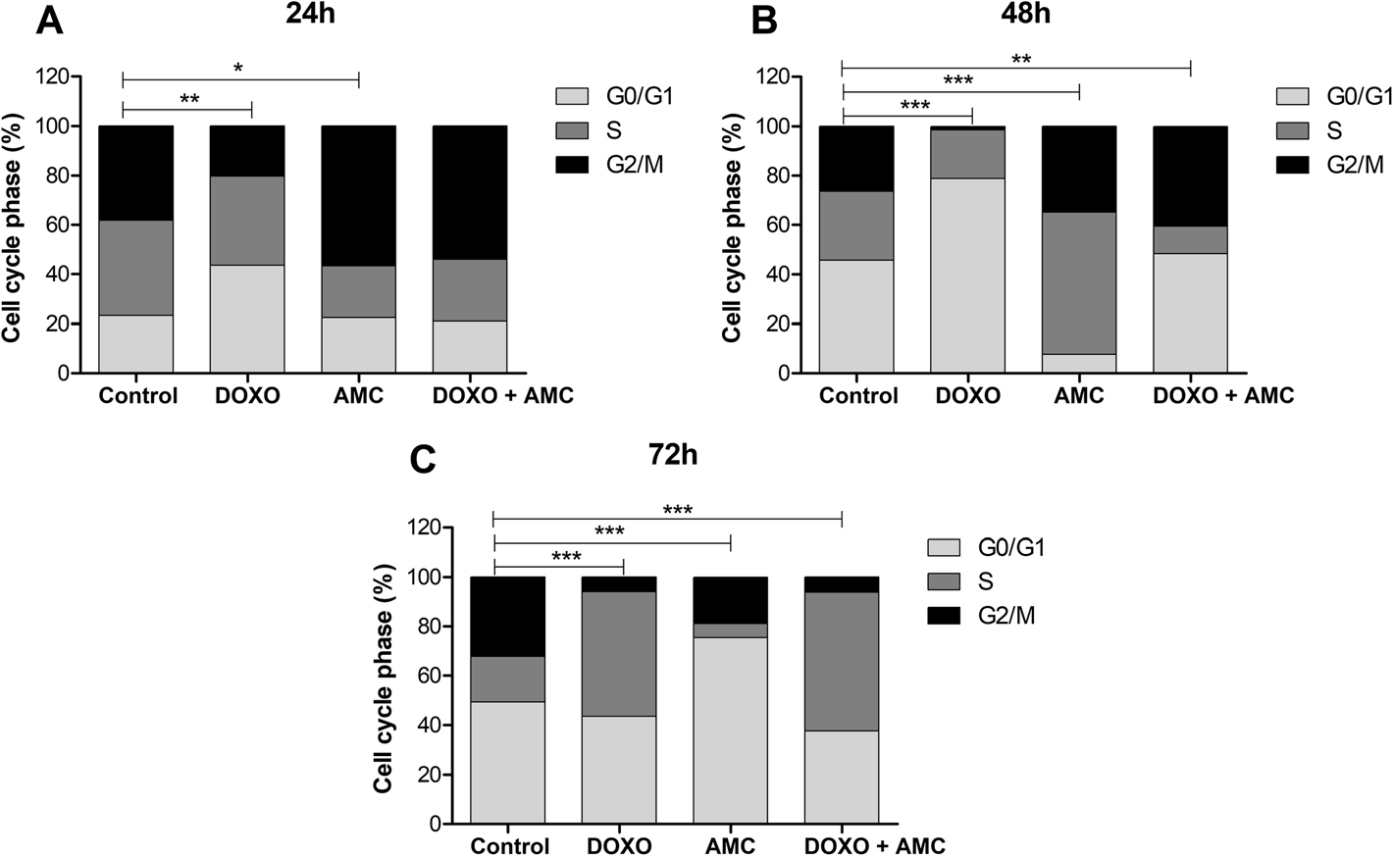
Cell cycle analysis at 24 (**a**), 48 (**b**) and 72 h (**c**), showing the percentage of cells in the interphase (G0/G1), DNA replication phase (S) and cell division (G2M). Data are averaged (*n* ≥ 03 ± SEM). Comparisions against control were performed by Chi-square analysis, *p < 0.05, **p < 0.01, ***p < 0.001

By cell cycle analysis it was possible to determine at which phase of the cycle the cells were during each treatment. In Group I, most of the cells were arrested in the GO/G1 phase (cellular interphase) throughout 48 h compared to control group. In Group II, the cells presented distinct peaks at each treatment time: at 24 h the greatest number of cells were expressed in G2/M, at 48 h in phase S and at 72 h in phase GO/G1. In Group III, at 24 h the cells were in G2/M, at 48 h the largest population was in GO/G1 and at 72 h in phase S (Fig. 3a-c).

### Flow cytometry results

**Fig. 4:**
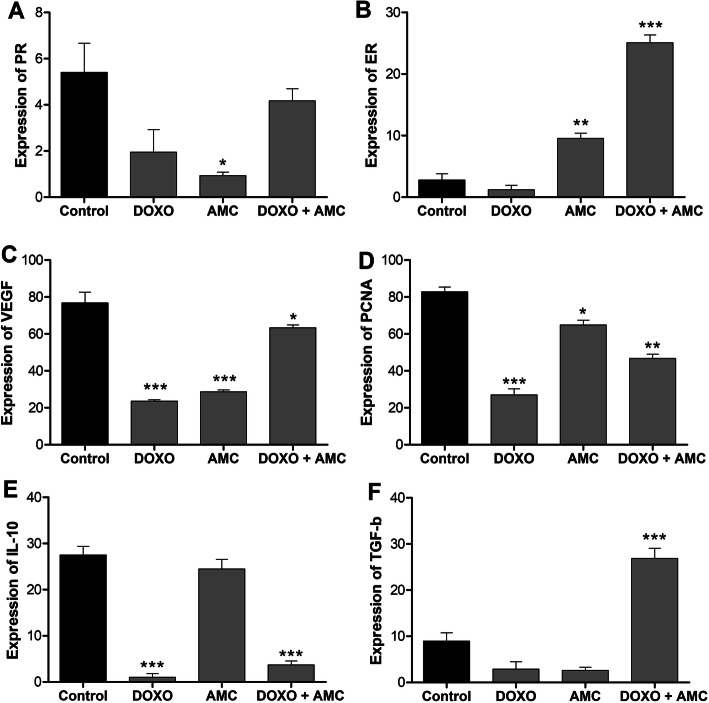
Cell marker expression (% of cells) by flow cytometry of control and cells treated with doxorubicin (DOXO, 10 µg/mL); amniotic membrane stem cells (AMCs); and DOXO + AMC. **a** Progesterone receptor (PR), **b** Estrogen receptor (ER), (**c**) VEGF, (**d**) PCNA-3, (**e**) IL-10, (**f**) TGF-β1. Data are averaged (*n* ≥ 04 ± SEM) of expression. (%). Comparisions against control were performed with one-way analysis of variance (ANOVA) as a Bonferroni post-hoc test, *p < 0.05, **p < 0.01, ***p < 0.001

The expression of proteins present in IPC-366 after the treatments was determined by flow cytometry analysis. Regarding the expression of PR, it was observed that control cells presented low expression (5.4%). However, Group II showed an even more significant reduction (0.9%). On the other hand, Group III showed an increase in PR expression, although it was not significant (Fig. 4a). Regarding ER, we observed an increase in its expression in Groups II and III, and in the association of DOXO with AMC the increase was approximately 10-fold higher than in the control group (Fig. 4b). In relation to the expression of VEGF, all treatment groups were effective in reducing its expression (Fig. 4c). A reduction in PCNA expression was observed in all treated groups (I–III; Fig. 4d). Regarding interleukin-10, we observed a reduction in its expression in Groups I and III (Fig. 4e). Finally, TGF-β was also analysed, and results showed low expression in control cells (8.9%), whereas in Group III, TGF-β1 expression was higher than in control (26.9%; Fig. 4f).

### Steroid hormone concentrations in culture medium

**Fig. 5:**
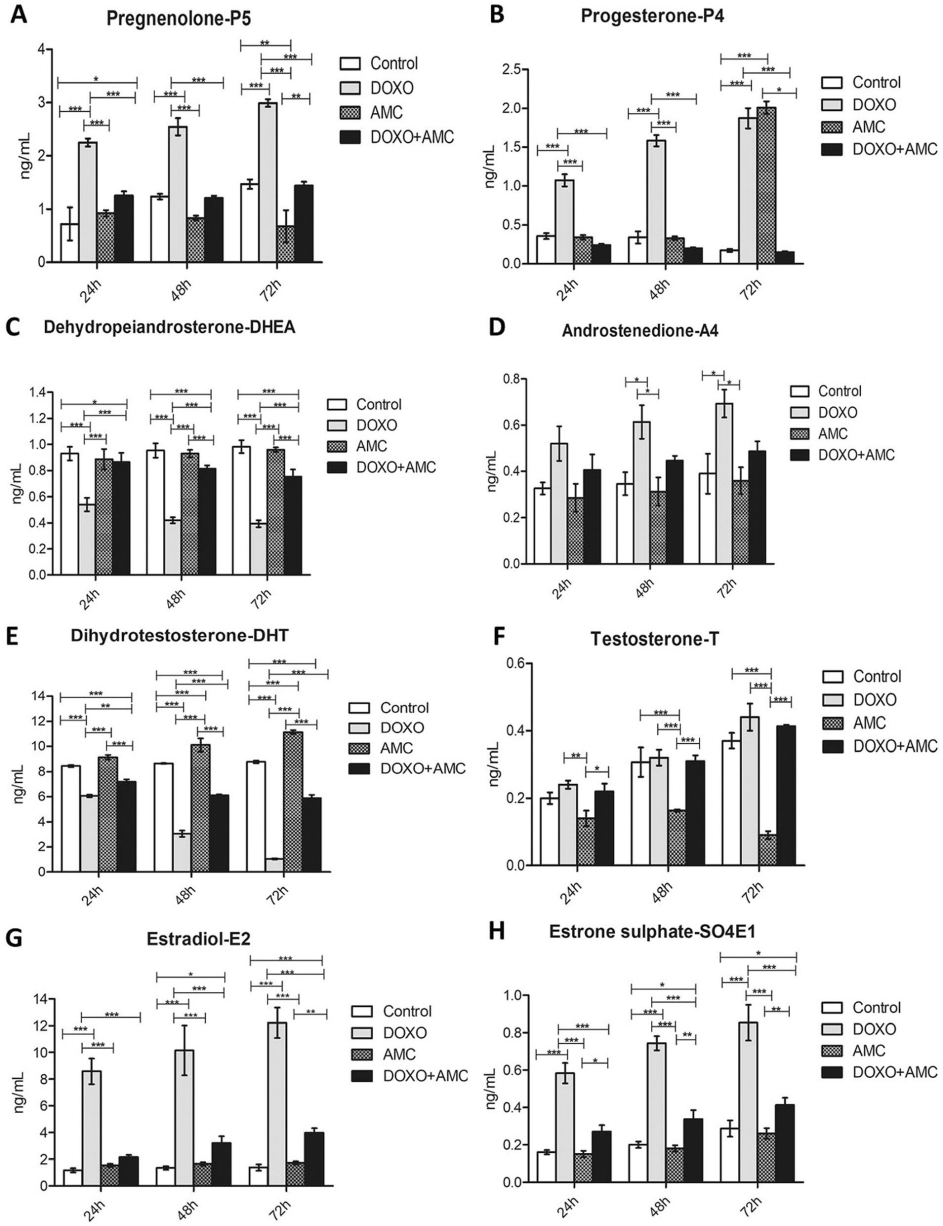
Steroid hormone levels in culture medium of IPC-366 control and treated cells. Graphs show hormone concentrations of pregnenolone (P5) (**a**), progesterone (P4) (**b**), dehydroepandrostenedione (DHEA) (**c**), androstenedione (A4) (**d**), dihydrotestosterone (DHT) (**e**), testosterone T (**f**), estradiol (**g**), estrone sulphate (SO4E1) (**h**) through the treatment period (24, 48 and 72 h). Treated groups (DOXO, AMC and DOXO + AMC, DOXO: 10 µg/mL) were compared to control group over the treatment period. The results presented as the mean ± SEM of 3 ndependent experiments (*p < 0.05; ** *p* < 0.01; *** *p* < 0.001). DOXO: doxorubicin; AMCs: canine amniotic membrane stem cells

The steroid hormone levels presented in the culture medium of IPC-366-treated cells were analysed by ELISA. In Group I it was possible to observe a significant increase (*p* < 0.05) in P4, A4, E2 and SO4E1 levels, and a reduction in DHEA and DHT levels (*p* < 0.05) in relation to the control group. In Group II, a significant increase (*p* < 0.05) was observed only in P4 and DHT levels, showing that the treatment was not sufficient to cause alterations in steroid hormone concentrations. Finally, treatment in Group III significantly reduced (*p* < 0.05) the androgens DHEA and DHT, whereas there was an increase in the estrogen levels (*p* < 0.05) E2 and SO4E1 (Fig. 5).

## Discussion

One of the most effective chemotherapeutic agents used for the treatment of haematological and solid tumours is doxorubicin [22]. Nevertheless, many types of tumour exhibit multidrug resistance, which may lead to a decrease in its effectiveness after a favourable initial response [23]. In our *in vitro* results we found that the optimal dosage of DOXO in treating IPC-366 cells was 10 µg/mL, resulting in a reduction in proliferation and cell growth of up to 72.46% after 72 h of treatment. This same dosage was used to treat the triple-negative adenocarcinoma cell line MDA-MB-231, where DOXO caused a 50% growth reduction after 24 h [24], demonstrating lower drug resistance when compared to IPC-366, which demostrated a growth reduction of 16.76% after 24 h. However, given that IPC-366 has been described as an aggressive tumour cell line [9], DOXO caused a reduction in cell growth, demonstrating that this drug can provide effective antitumor activity in canine mammary cancer. Currently, resistance to chemotherapy represents the biggest obstacle to success in the treatment of human and canine breast cancer [25].

The IPC-366 control cells presented an epithelial morphology as described by Caceres et al., 2015 [9]. In this work, during treatment with AMCs it was possible to verify that, despite all the factors released by the AMCs, they did not influence the cell morphology; however, after treatment with DOXO they did show cellular alterations, such as cell membrane rupture and a rounded shape, indicating cellular apoptosis. This is in accordance with other studies that tested the effect of this drug on tumour cells [26].

Tumor cells are characterised by uncontrolled growth and cell proliferation [27]; one mechanism of ascertaining the effectiveness of anticancer treatments is their role in interrupting the cell cycle [28]. Doxorubicin is a non-specific agent of the cell cycle, acting in the phases of division and rest. However, its main cytotoxic action is observed during the S phase of the cell cycle, by altering the DNA structure and producing free radicals that help fight cancer [29]. These effects were found in this study for Groups I and III, where the majority of cells were found in the S phase after 72 h of DOXO treatment. However, in Group II, treatment with AMCs, it was found that the cells were in the GO/G1 interphase. This can be explained by the release of cytokines by mesenchymal stem cells. These cytokines are capable of inducing cell cycle arrest in tumour cells [30].

Sex steroid hormones, especially estrogens, play a key role in mammary carcinogenesis in mammals, including female dogs [31]. An important predictor for breast cancer is the evaluation of the ER and PR expression profile, which allows clinicians to predict the response of breast cancer to treatment [32]. If a higher ER and PR expression is observed in the tumour, there is a greater probability of response to hormone therapy [33]. Therefore, the response rate to the treatment of patients classified as ER(–) and PR(–) is only 10%. This rate increase to 40% [34] in ER(+) and PR(–) patients. IPC-366 cells were characterised as negative for ER and PR expression, and after treatment with DOXO and AMCs (Group III), a reversal in their expression was observed. The cells returned a positive phenotype for ER with a percentage expression of 25% and remained negative for PR with a percentage of 4.17%.

In addition to the hormone receptors, other proteins were also evaluated, such as the VEGF involved in tumour angiogenesis. Results showed a lower rate of expression in the DOXO treatment, demonstrating a beneficial effect of the chemotherapy. The inhibition of VEGF reduces the abnormality of the vessels and increases the tumour permeability to chemotherapy [35]. This reduction was also observed for the protein PCNA, which was highly expressed in the IPC-366 cell line and, after all treatments, a significant reduction in its expression was observed. This reduction in PCNA expression with DOXO treatment can be explained by its role in the inhibition of topoisomerase II, which induces DNA breakage, inhibiting its replication [36]. Since amniotic membrane-derived cells have antiproliferative effects, they can arrest the cell cycle in the G0/G1 phase due to unknown factors [37]. These factors, produced by amniotic stem cells, can regulate the expression of cancer cell proteins associated with the cell cycle, such as cyclin-dependent kinases (CDK2, CDK4, CDK6) and cyclins (cyclin D2, cyclin E1, cyclin H).

Our analyses showed that the lowest expression of the interleukin IL-10 occurred when DOXO was used, whether or not associated with AMCs. This drug contributed to the reduction of the inflammatory and immunosuppressive responses, promoting a favourable microenvironment for an immune response to the tumours. This is in accordance with Nugroho et al. (2012) [38], who verified the immunosuppressive effects of DOXO.

Another molecule involved in signalling pathways in breast cancer is TGF-β, which can induce epithelial–mesenchymal transition; its expression can be influenced negatively by oestrogen levels. ER-α inhibits TGF-β signalling by decreasing Smad protein levels. High estrogen levels cause a suppression in the levels of TGF-β1 [39]. This could also be observed in our treatments, since Groups I and II, which did not present estrogen expression, were also absent for TGF-β, unlike Group III, which presented expression for estrogen and consequently for TGF-β.

On the other hand, the normal activity of the normal cells depends on the perfect integration between its metabolic pathways, which is mainly controlled by the steroid hormones [40]. These hormones, among other factors, induce or promote carcinogenesis by stimulating cellular proliferation [41]. It is known that there are two steroidogenic pathways for the production of steroid hormones, both presenting cholesterol as a precursor molecule [42]. First, cholesterol is converted to P5, which is the first hormone of the steroidogenic cascade and precursor of the hormones DHEA and P4. In one of the steroidogenic pathways, androgens are formed from the conversion of P5 into DHEA, which leads to the formation of A4. On the other pathway, P5 is initially converted to P4, which is consequently converted to A4, an androgen precursor. The first pathway is known as the Δ5 pathway, and the second the Δ4 pathway [43]. A4 can be converted into T, which is the precursor hormone of DHT. Indeed, A4 can be converted directly to estrogens by the conversion of A4 to estrone (E1) and subsequently in SO4E1. Finally, testosterone can also give rise to estrogens, resulting in the production of E2 [44]. Therefore, neoplastic cells can experience an imbalance in the steroidogenic pathways. Because of this, hormone production is one of the targets of therapies for the treatment of various neoplasms, especially breast cancer [45, 46]. For this reason, our study evaluated the interference of treatments in the levels of the main steroid hormones.

In Group I, treated with DOXO, we observed that no interference occurred in estrogen pathways, since E2 and SO4E1 levels were increased at the end of the treatment period. However, DHEA was at low levels, indicating inhibition of the Δ5 pathway, which is the preferred steroidogenic pathway in IBC [47]. Other *in vitro* studies also measured these hormones after treatment with androgen inhibitors with similar results to our DOXO treatment by choosing the Δ5 pathway [48]. The results obtained by hormonal analysis, where the presence of oestrogen production was verified, are due to the data obtained in the analysis of flow cytometry. The absence of ERs was observed and, although the steroid hormones could enter the cells through the membrane [49], they could be accumulated in the medium and thus promote no functional effects on IPC-366 cells. Steroid hormone receptors depend on intracellular transcription factors that mediate their biological actions [50]. Besides that, receptors are responsible for hormone signalling transduction to the target genes, interacting with specific DNA sequences and several regulatory proteins including activators or co-repressors [45]. Therefore, the absence of receptor expression causes the continuity of malignancy in this type of carcinoma.

In Group II, treatment with AMCs did not interfere at all stages of the steroidogenic metabolism of IPC-366 cells. The results suggest that P5 reduction may have occurred because of its total conversion to progesterone after the first 48 h of treatment; similar results were observed on T levels, which were reduced dramatically after 24 h of treatment, being fully converted into DHT. This DHT accumulation may suggest that AMC treatment may be effective in reducing the proliferation of breast cancer tumour cells, since this is the most potent androgen for this function [51], as other studies have observed for MCF-7, another breast cancer cell line [52].

**Fig. 6:**
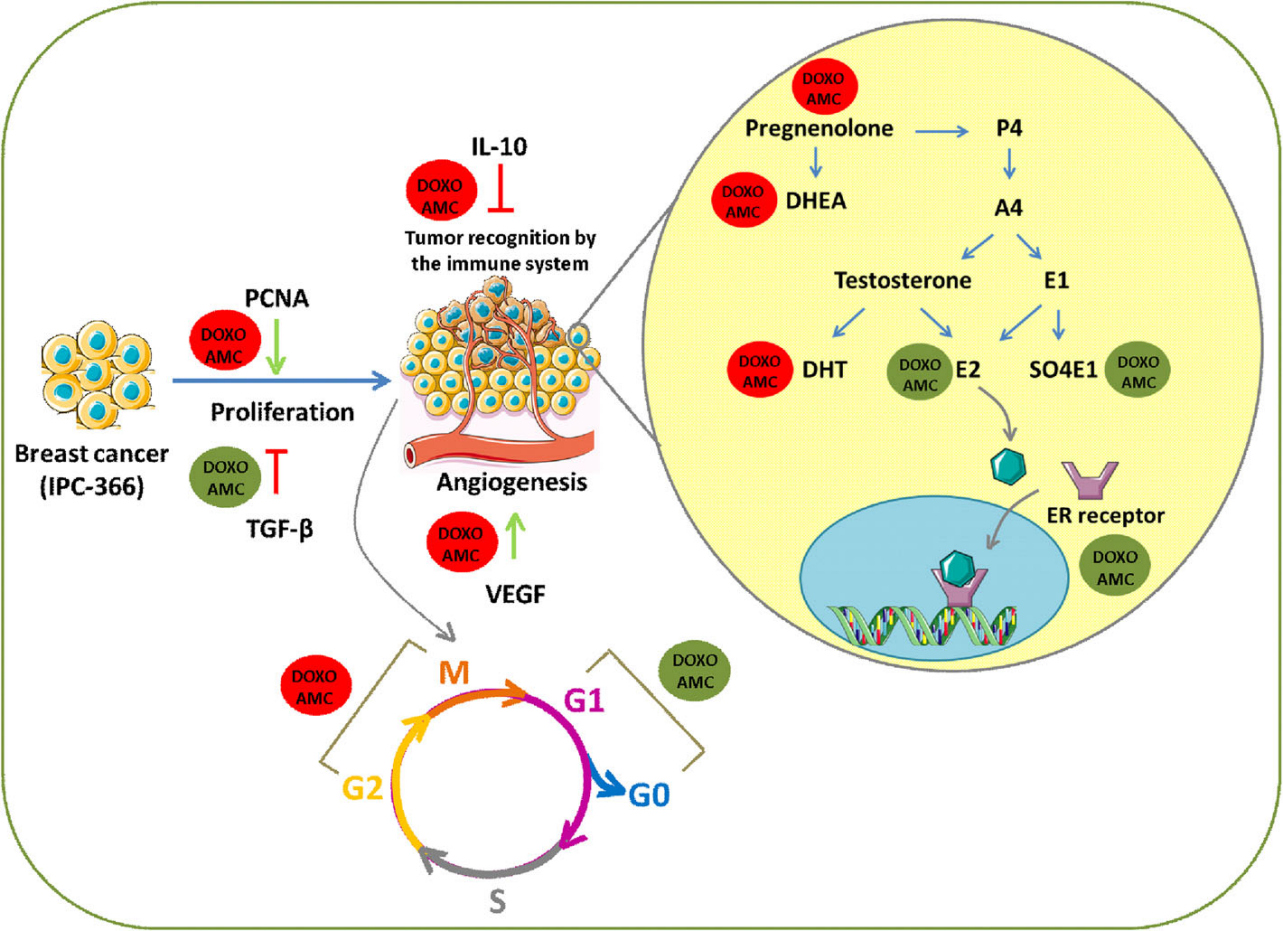
Scheme of the main effects of the treatment with doxorubicin (DOXO) and canine amniotic membrane cells (AMCs) in IPC-366 cells. The proliferation and angiogenesis of IPC-366 cells were increased due to the influence of PCNA, TGF-β1 and VEGF; however, treatment with DOXO + AMC association reversed this situation. Moreover, the treatment with the DOXO + AMC decreased IL-10 levels, which may have allowed tumour cells to be recognised by the components of the immune system and to decrease the inflammatory process. In addition, after treatment with DOXO + AMC there was an increase of cells in the G1/G0 phase and decrease in those in the G2/M phase due to a reduction in cell duplication. Furthermore, after treatment with the combination, there were reductions in the levels of P5, DHEA and DHT, while there were increases in E2 and SO4E, important steroid hormones in the steroidigenic pathway. We also observed that there was an increase in ER expression, which can be used as a therapeutic target P4: progesterone; A4: androstenedione; E1: estrone

Finally, in Group III (association of DOXO and AMCs), the treatment did not significantly alter the levels of the hormones of the Δ4 pathway, probably due to the low availability of P5 as a substrate. DHEA levels suffered a significant reduction, as occurred in Group I, which leads us to suppose that the AMCs exerted a modulatory effect on the hormonal production of the Δ4 pathway (Fig. 6).

## Conclusions

The association of DOXO and AMCs has been shown, for the first time, to be an alternative and effective treatment for triple-negative tumours, since we found an increase in ERs, resulting in cells that could be susceptible to anti-estrogen therapy, allowing the application of hormone therapy in these tumours.

## Data Availability

The datasets generated and/or analysed during the current study are not publicly available due to the data of characterisation of AMCs being at the writing stage of the manuscript for publication, but are available from the corresponding author on reasonable request.

## Abbreviations

A4: Androstenedione
AMC: Canine amniotic membrane stem cells
DHEA: Dehydropeiandrosterone
DHT: Dihydrotestosterone
DOXO: Doxorubicin
E2: Estradiol
ER: Estrogen receptor
IBC: Inflammatory breast carcinoma
IPC-366: Triple-negative canine inflammatory mammary carcinoma cell line
P4: Progesterone
P5: Pregnenolone
PI: Propidium iodide
PR: Progesterone receptor
SO4E1: Estrone sulphate
T: Testosterone
